# Lanthanide doped nanoparticles for reliable and precise luminescence nanothermometry in the third biological window

**DOI:** 10.1039/d2na00941b

**Published:** 2023-05-23

**Authors:** Ana C. C. Soares, Tasso O. Sales, Erving C. Ximendes, Daniel Jaque, Carlos Jacinto

**Affiliations:** a Group of Nano-Photonics and Imaging, Instituto de Física, Universidade Federal de Alagoas 57072-900 Maceió-AL Brazil cjacinto@fis.ufal.br; b Nanomaterials for Bioimaging Group (nanoBIG), Departamento de Física de Materiales, Facultad de Ciencias, Universidad Autónoma de Madrid Madrid 28049 Spain daniel.jaque@uam.es; c Nanomaterials for Bioimaging Group (nanoBIG), Instituto Ramón y Cajal de Investigación Sanitaria Hospital Ramón y Cajal Madrid 28034 Spain

## Abstract

In recent years, infrared emitting luminescent nanothermometers have attracted significant attention because their potential for the development of new diagnosis and therapy procedures. Despite their promising applications, concerns have been raised about their reliability due to the spectral distortions induced by tissues that are present even in the commonly used second biological window (1000–1370 nm). In this work, we present an innovative solution to this issue by demonstrating the effectiveness of shifting the operation range of these nanothermometers to the third biological window (1550–1850 nm). Through experimental evidence using ytterbium, erbium, and thulium tri-doped CaF_2_ nanoparticles, we demonstrate that luminescence spectra acquired in the third biological window are minimally distorted by the presence of tissue, opening the way to reliable luminescence thermometry. In addition, advanced analysis (singular value decomposition) of emission spectra allows sub-degree thermal uncertainties to be achieved.

## Abstract 2

The use of luminescent nanoparticles working in the third biological window opens the possibility of reliable and accurate sub-tissue thermal sensing.

## Graphical abstract

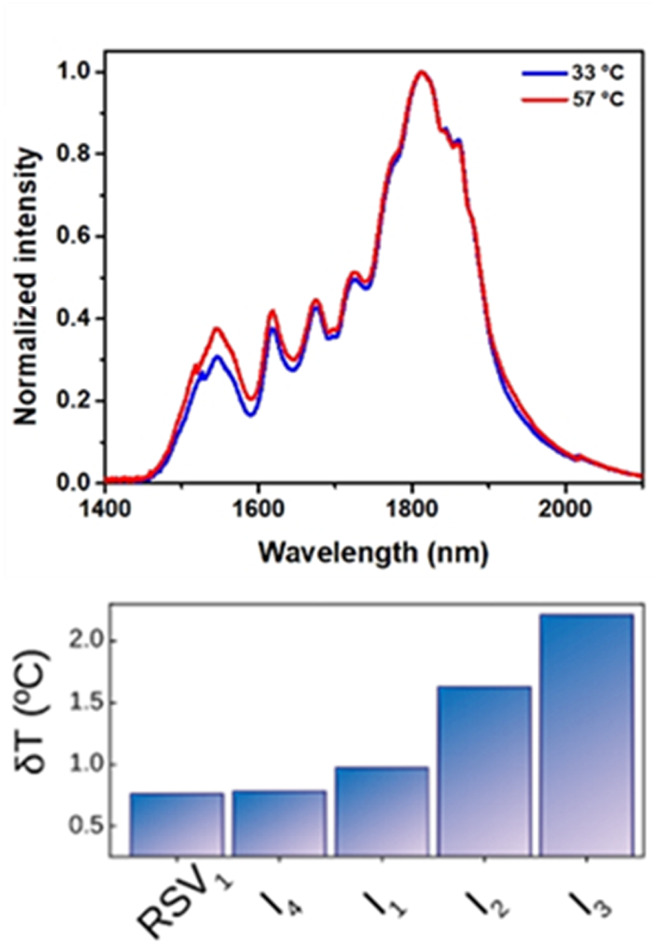

## Introduction

Temperature governs and controls physiological processes.^1,2^ In recent years, different nanomaterials have been proposed as thermal reporters,^3–5^ and, despite their applicability in a diversity of fields, they have found some limitations when being applied in biomedicine. For instance, thermal measurements in living systems should be achieved in a remote way in order to interfere minimally with the normal behaviour of the system. In order to achieve such remote thermal monitoring luminescent nanothermometers (LNThs) have emerged as a potential solution.^5,6^ LNThs are luminescent nanoparticles whose emission is temperature dependent so that thermal readout can be achieved from an adequate analysis of their emission. Thermal readout can be achieved from the analysis of one or more spectroscopic parameters such as the peak position, linewidth, intensity ratio or polarisation state.^7–9^

LNThs have been able to provide thermal readouts inside cells and living animals.^7,8^ Indeed, LNThs have already been used for sub-degree thermal reading in tissues, for early detection of incipient diseases such as ischemia and tumours, for tissue diagnosis and for providing thermal control in photothermal therapies at the *in vivo* level.^7,9,10^ In spite of this recent success, the *in vivo* application of luminescence thermometry needs to overcome some inherent limitations that are related to the optical extinction of tissues.

On one hand, the strong attenuation of visible light in tissues makes it difficult to access internal organs optically.^11^ This limits the potential of visible-emitting LNThs. Indeed, *in vivo* luminescence thermometry requires working in the near-infrared (NIR) spectral range due to the lower absorption and scattering of light in this spectral range. Traditionally, NIR is divided into three biological windows (BWs), where optical extinction is minimum: the first (BW-I) extending from 650 up to 950 nm, the second (BW-II) ranging from 1000 up to 1370 nm and the third (BW-III) from 1550 up to 1870 nm.^12,13^ BW-I has the drawback of interference with autofluorescence;^14,15^ however, the absence of water absorption in this spectral range makes it ideal for laser excitation.^16,17^ Operating within BW-II results in an improved signal-to-noise ratio, a reduced (but not negligible) autofluorescence background and availability of cost-effective fluorescence cameras.^18,19^ All these together have made BW-III very popular for pre-clinical fluorescence imaging. Finally, BW-III offers advantages such as the complete absence of autofluorescence and minimum tissue extinction but it is not extensively explored due to the absence of accessible fluorescence cameras working in this spectral range.^13,20,21^ As a consequence of all these pros and cons most of the work on *in vivo* fluorescence imaging and thermometry has been traditionally developed by using probes working in BW-I,^22–24^ although during the last few years the use of optical probes working in BW-II has become more and more popular. A reduced number of studies have demonstrated the suitability of optical probes working in BW-III for high penetration *in vivo* imaging.^21,25–30^

Tissue-induced light extinction not only affects the depth achievable by LNThs but also impacts their reliability.^31–39^ When a given tissue has a flat extinction spectrum, meaning it lacks any significant peaks or valleys within a certain wavelength range, the light that passes through it will retain its spectral shape (it will retain the relative intensities between emission lines). Hence, while there will be an overall reduction in intensity, the shape of the spectrum will remain the same. This property becomes particularly useful in luminescence thermometry. To grasp this concept, let's consider a scenario where a LNTh is located within a medium that has an extinction coefficient with thermal and wavelength dependencies described using *μ*(*λ*,*T*). According to the Beer–Lambert law, the luminescence intensity detected at wavelength *λ* after passing through the medium at a temperature *T* is given by:

1 *I*_det_(*λ*,*T*) = *I*_o_(*λ*,*T*)e^−*μ*(*λ*,*T*)*L*^

**Fig. 1:**
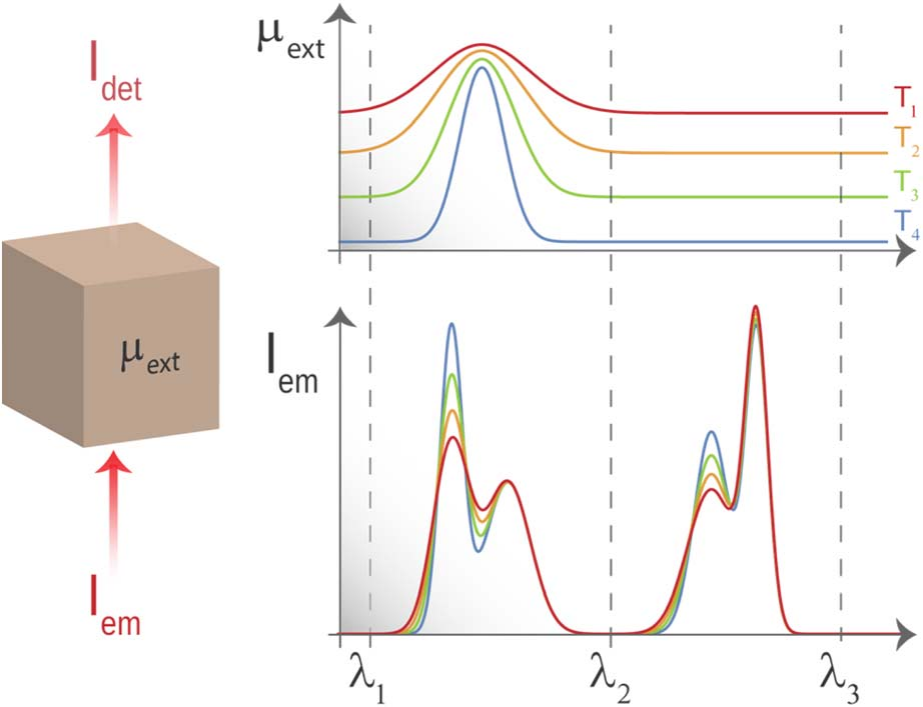
The light that propagates through a tissue is affected by absorption and scattering processes that result in an effective reduction of its intensity. This reduction is best described by what is usually called the extinction (or attenuation) spectrum. It is observed that, for thermometry based on fluorescence intensity ratios, the performance is improved when the ratio is calculated between intensities found within wavelength ranges where the attenuation spectrum is flat.

Thus, the ratio between the detected intensities at *λ*_i_ and *λ*_f_ is given by:2
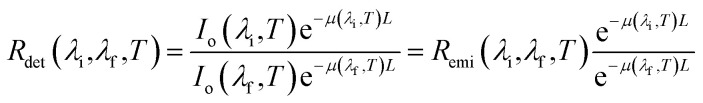
which means that if *λ*_i_ and *λ*_f_ are within an interval in which the extinction coefficient of the surrounding medium varies with the wavelength (range [*λ*_1_, *λ*_2_] schematically represented in Fig. 1), it is mandatory to know the value of *μ* at both *λ*_i_ and *λ*_f_ to properly convert a calibration made only with the ratio between the emitted intensities (*R*_emi_) into the one that is valid even under the presence of the propagating medium. This limits the potential application of luminescence thermometry as it requires the precise knowledge of how the extinction of a medium depends on both wavelength and temperature. In the case of tissues, this is far from being simple.

However, if *λ*_i_ and *λ*_f_ are within the spectral range in which the extinction coefficient of the surrounding medium is flat (range [*λ*_2_, *λ*_3_] schematically represented in Fig. 1), the detected ratio becomes:3
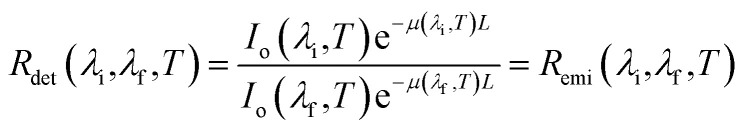
*i.e.*, the insertion of a luminescent thermometer into the medium does not alter the calibration of the thermometer based on the ratio between emitted intensities. Thus, if we could find a spectral region where that was generally valid for biological tissues, the issue of tissue-induced spectral distortions would be avoided and the thermal readout provided by ratiometric nanothermometers would also become reliable.

**Fig. 2:**
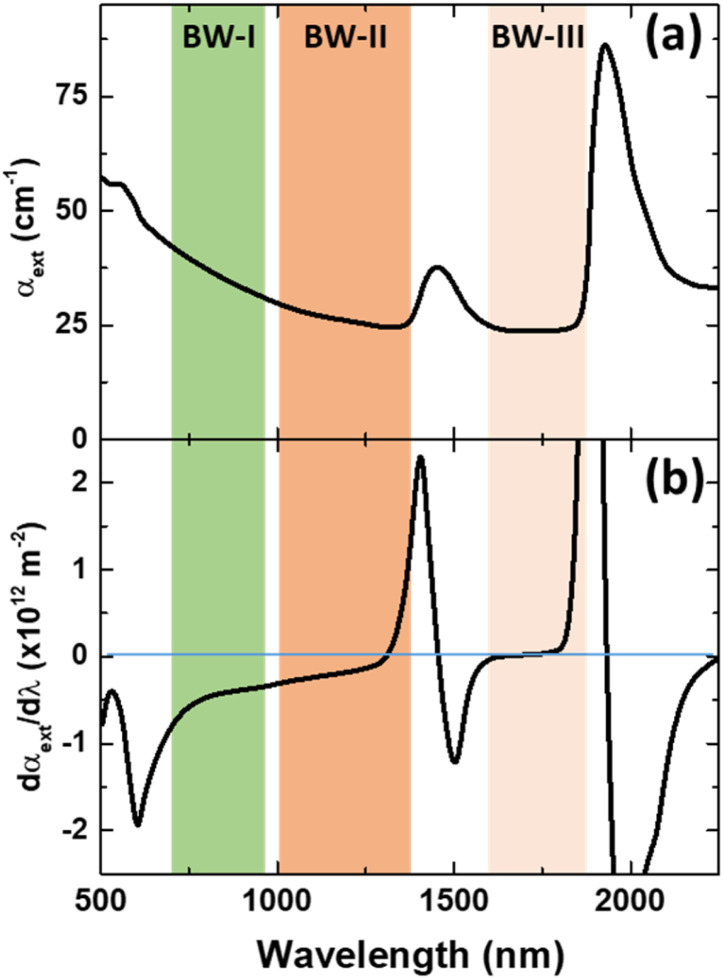
(a) Extinction coefficient of human (prostate) tissue. The spectral extension of the three biological windows is indicated. Data obtained from ref. 40. (b) Derivative of the absorption coefficient of human tissue with respect to the wavelength as calculated form (a).

Luckily, when analysing the extinction coefficient, *μ*, of representative human tissues (Fig. 2(a), as obtained from ref. 40) in the 600–2250 nm spectral range, it is found that in the 1590–1860 nm region, *μ* is approximately constant. And this range is found within what is generally described as BW-III.^12^ To further demonstrate this, Fig. 2(b) shows the derivative of the extinction coefficient as calculated from Fig. 2(a) showing how this remains close to zero in a great portion of BW-III. As explained above, this means that thermometry based on the luminescence intensity ratio can be made reliable. Despite that there are already some studies reporting on LNThs operating in BW-III,^25,29,41–44^ none of them have explored the possible presence/absence of tissue-induced spectral distortions.

In this work we have investigated the use of lanthanide doped nanoparticles for thermal sensing in BW-III. In particular we have evaluated the performance of Yb^3+^, Er^3+^ and Tm^3+^ doped CaF_2_ nanoparticles (NPs) as LNThs in BW-III. CaF_2_ NPs have been selected as host materials due to their low effective phonon energy (280 cm^−1^ that ensures low nonradiative decay probabilities), broad transparency window (from UV to IR), simple synthesis route, and low toxicity in biological systems.^45–48^ Experiments have been conducted to demonstrate the suitability of Tm^3+^ emissions in BW-III for reliable (distortion-free) luminescence thermometry. In addition, we have also explored the potential use of advanced analysis to improve the thermal uncertainties of the thermal readouts provided by CaF_2_ NPs.

## Experiments

### Synthesis of nanoparticles

For the synthesis of the nanoparticle (CaF_2_:10Yb^3+^/6Er^3+^/1Tm^3+^), an amount of 0.83 mmol of CaCl_2_, 0.1 mmol of YbCl_3_, 0.06 mmol of ErCl_3_ and 0.01 mmol of TmCl_3_ were added in a 100 ml round bottom single neck flask containing 20 ml of Milli-Q water and the solution was heated at 90 °C, approximately, under constant stirring. Subsequently, 2 mmol of a NH_4_F solution was injected into the mixture dropwise and kept at 90 °C for 1 h under vigorous stirring. After this time and after being cooled down to room temperature naturally, white precipitates were collected by centrifugation and washed with Milli-Q water three times. The resulting product was dried at 60 °C in an ambient atmosphere for 48 h and finally heat treated at 500 °C for 3 h. We did not simply choose these rare-earth ion concentrations, but a detailed study was carried out in which we concluded that this sample is the best in terms of luminescence relative to the investigated bands.

### Experimental apparatus

**Fig. 3:**
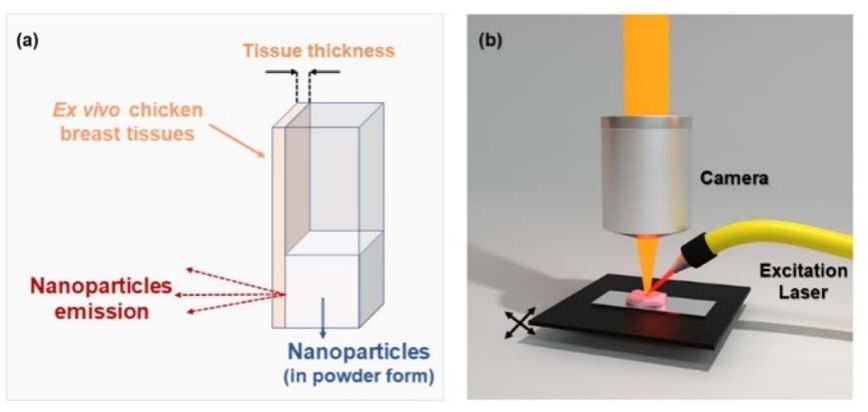
Schematic representations of the experimental setup used for spectral distortions induced by tissue. (a) Representation of *ex vivo* tissue disposition and used nanoparticles. (b) Illustration of the experimental system used for spectral distortion of the Yb–Er–Tm CaF_2_ NPs induced by *ex vivo* tissue.

The luminescence measurements were performed by using a fluorometer (NanoLog, Horiba) coupled to an R928P or R5509-73 photomultiplier tube or a liquid nitrogen refrigerated solid state extended InGaAs detector. The 10Yb^3+^/6Er^3+^/1Tm^3+^ co-doped CaF_2_ NPs were optically excited using a Lumics Laser operating at 940 nm, which was focused on the sample. For thermal analysis, Peltier plates connected to a voltage source were used as heating systems, and the electrical voltage was manually adjusted at the source to work within the physiological temperature range. The measurements for biological applications were made using *ex vivo* chicken breast tissue. Tissue-induced spectral distortions were investigated by placing the chicken breast in front of the cuvette containing the NPs; the fluorescence signal was collected using an optical fiber and dispersed to a monochromator, as shown in Fig. 3(a). The luminescence spectra/images were collected using a confocal fluorescence microscope (LabRam Evolution UV-VIS-NIR) coupled with a Symphony II detector and focused with a 20 × long working distance microscope. In this case, we placed the biological tissue under the mobile platform and scanned it, as illustrated in Fig. 3(b).

## Results and discussion

**Fig. 4:**
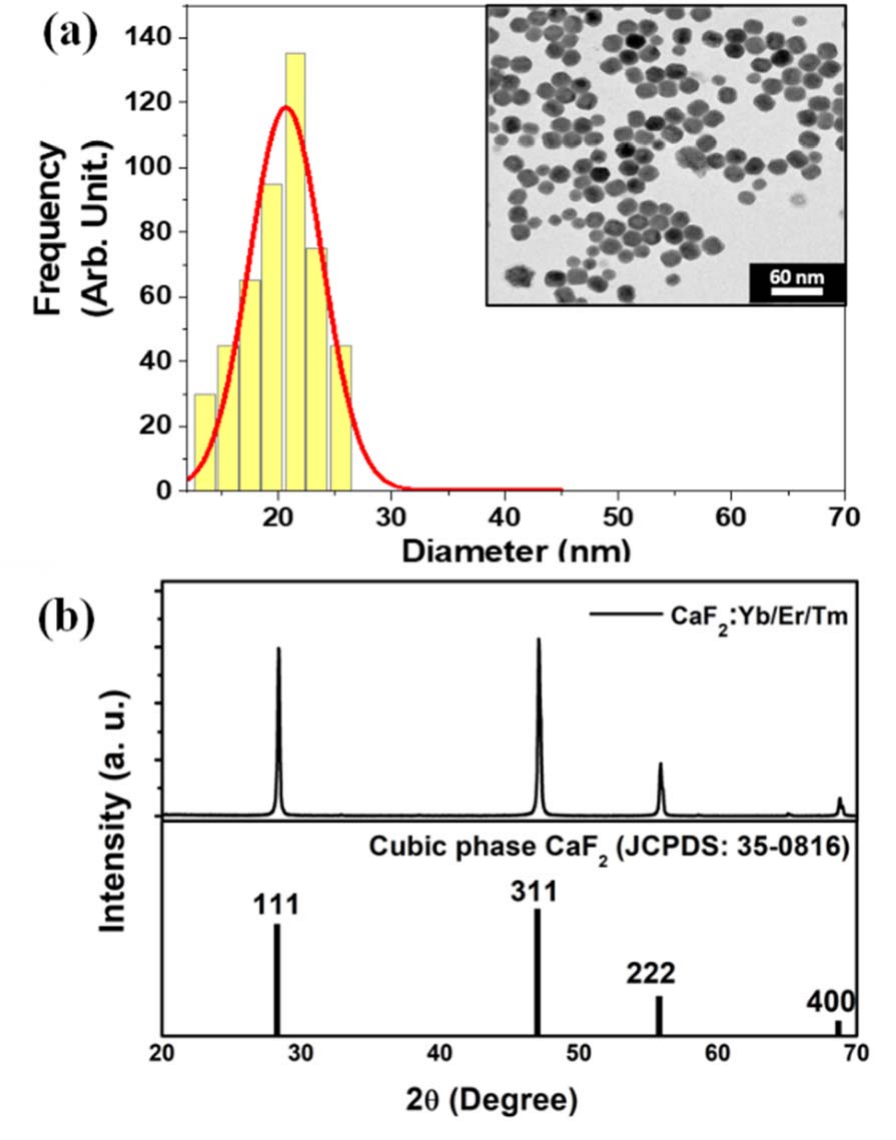
(a) Size distribution for the CaF_2_:Yb^3+^/Tm^3+^/Er^3+^ nanoparticles. The inset shows a TEM image of these nanoparticles. The bar size is 60 nm. (b) X-ray powder diffraction patterns of CaF_2_:Yb^3+^/Tm^3+^/Er^3+^ nanoparticles (top) and the standard XRD pattern (bottom).

The inset of Fig. 4(a) shows a characteristic transmission electron microscopy (TEM) image of the CaF_2_:Yb/Tm/Er NPs utilized in this work. The size histogram that has been calculated from the statistical analysis of the TEM image is also included in Fig. 4(a), from which fitting to lognormal distributions resulted in an average diameter of 20.8 ± 0.3 nm with a width of 7.5 nm (*R*-square = 0.93 was obtained). A typical XRD pattern of CaF_2_:Yb/Tm/Er NPs is shown in Fig. 4(b)-top. The sharp diffraction peaks in the pattern in Fig. 4(b)-bottom can be indexed as pure cubic-phase CaF_2_, which is in good agreement with the standard XRD pattern JCPDS 35-0816 (Fig. 4(b)-bottom). No other impurity peaks were identified, which indicates formation of pure cubic-phase CaF_2_ structures with a highly crystalline nature. Compared with the standard peaks, all the diffraction peaks of pure cubic-phase CaF_2_ are barely shifted, because the ionic radius of Ca^2+^ is close to the dopant ionic radius of the ions Yb^3+^ and Er^3+^.

**Fig. 5:**
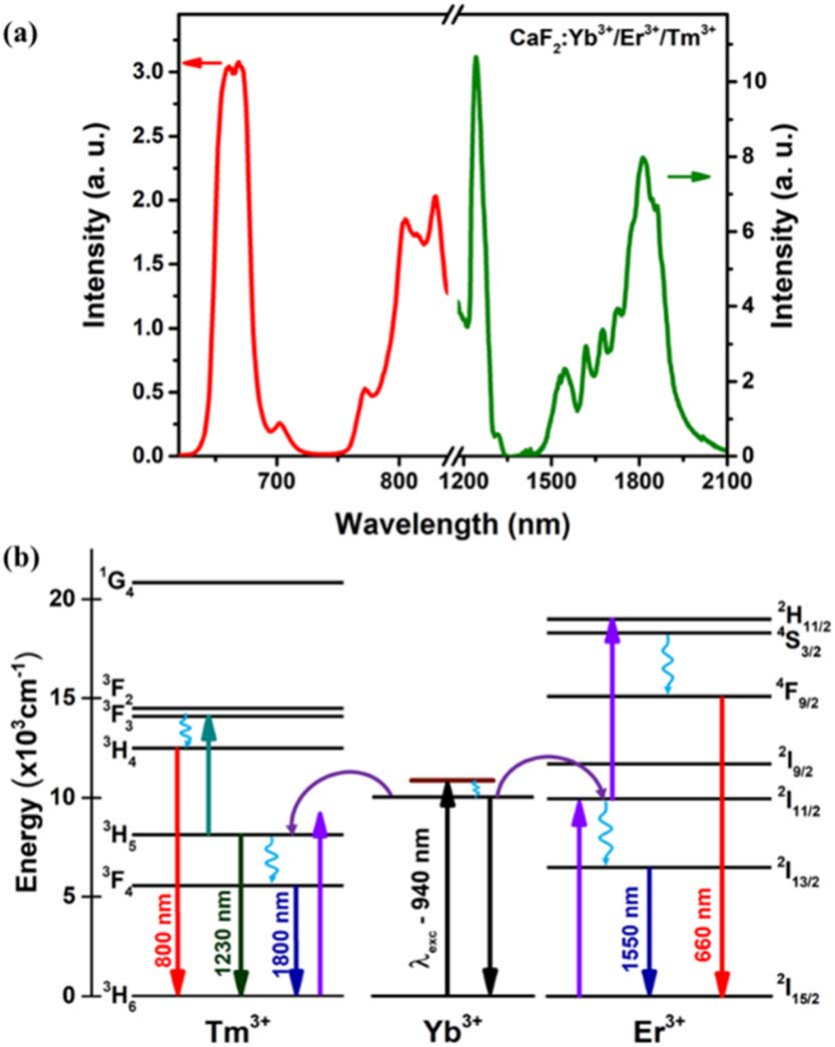
(a) Emission spectrum of Yb^3+^/Er^3+^/Tm^3+^ tri-doped CaF_2_ NPs under 940 nm excitation at room temperature in the I-BW (red), II-BW (left peak of the green spectrum) and III-BW (right peak of the green spectrum). (b) Simplified energy scheme of the Yb^3+^, Er^3+^ and Tm^3+^ ions, showing excitation and emission decays (full lines), nonradiative decays (wavy lines), and the energy transfer process (curved lines).

At room temperature and under 940 nm excitation, the Yb–Er–Tm triply doped CaF_2_ NPs present four characteristic emission bands at around 660, 1230, 1550 and 1800 nm, as shown in Fig. 5(a). These luminescence bands lie within the three BWs as defined previously. In addition, the laser excitation at 940 nm (in BW-I) is sufficiently far from the water absorption peak (∼980 nm) so that heating is avoided. The Yb^3+^ ions act as sensitizers absorbing the 940 nm radiation through the ^2^F_7/2_ → ^2^F_5/2_ transition and by means of energy transfer (ET) both Er^3+^ (^4^I_11/2_) and Tm^3+^ (^3^H_5_) ions are excited. Multiphonon nonradiative decays populate the ^4^I_13/2_ energy level of Er^3+^ ions from which the emission at around 1550 nm is produced (^4^I_13/2_ → ^4^I_15/2_ transition). A second photon excites the Er^3+^ ions to the ^4^S_3/2_ level through a second ET from the Yb^3+^ to Er^3+^ ions, from which multiphonon decay occurs to the ^4^F_9/2_ level, promoting the emission at around 660 nm (^4^F_9/2_ → ^4^I_15/2_ transition). The ^3^H_5_ → ^3^H_6_ radiative decay of Tm^3+^ ions gives rise to the emission around 1230 nm. Multiphonon decay from the ^3^H_5_ level populates the ^3^F_4_ state, from which the emission at around 1800 nm is generated (^3^F_4_ → ^3^H_6_ transition). Excitation of Tm^3+^ ions from the ^3^H_5_ level to the ^3^F_2_ state occurs thanks to energy transfer from Er^3+^ ions. Nonradiative transitions from the ^3^F_2_ state populate the ^3^H_4_ energy level from which the emission at around 800 nm is generated (see Fig. 5(b)).

**Fig. 6:**
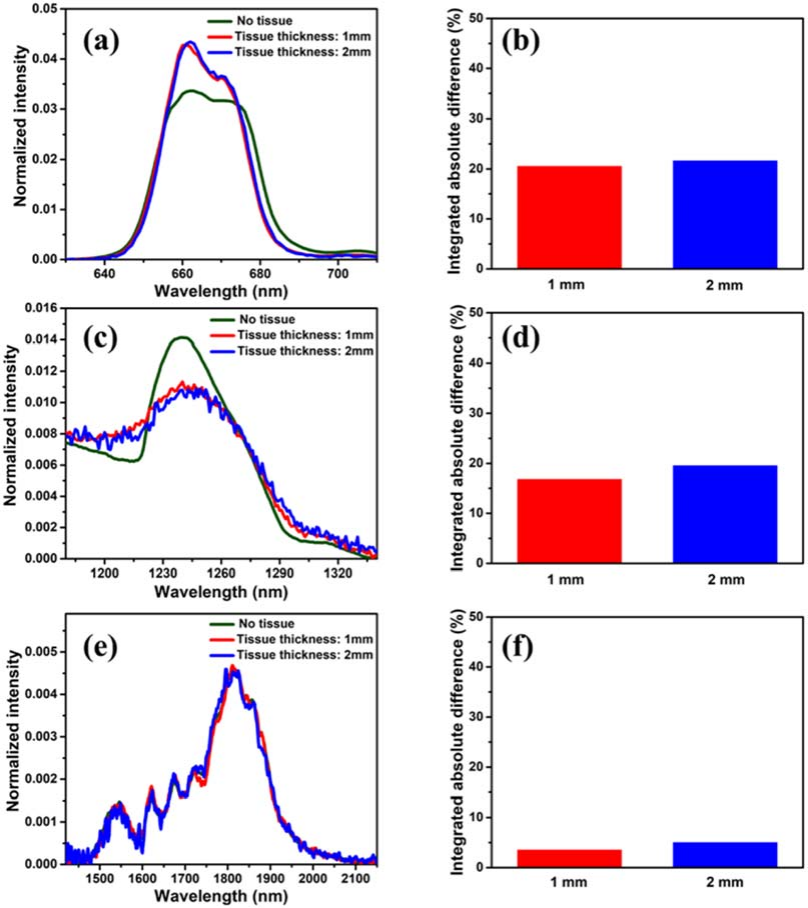
Emission spectra of Yb^3+^/Er^3+^/Tm^3+^ tri-doped CaF_2_ NPs under 940 nm excitation at room temperature without and with 1.0 and 2.0 mm of tissue in the (a) III-BW, (c) II-BW, and (e) I-BW. The percentage difference in the spectral shape for tissues' thickness with 1.0 mm (red) and 2.0 mm (blue) in the (b) III-BW, (d) II-BW, and (f) I-BW.

For the sake of evaluating the performance of CaF_2_:Yb^3+^/Er^3+^/Tm^3+^ NPs for subtissue applications, we have investigated the spectral distortions induced in the different emission bands of CaF_2_:Yb^3+^/Er^3+^/Tm^3+^ NPs by chicken breast tissues with thicknesses of 1.0 and 2.0 mm (red and blue curves in Fig. 6(a), (c) and (e)). It is possible to notice that the emission bands lying within BW-I and BW-II present larger distortions when compared to those induced in the III-BW. Indeed, the Tm^3+^ emission at around 1700 nm practically maintains its spectral shape even in the presence of a 2 mm thick tissue. To quantify the tissue-induced spectral distortions, the percentage difference (*σ*) in the spectral shape was evaluated according to a parameter previously defined by Ximendes *et al.*^49^ as:4
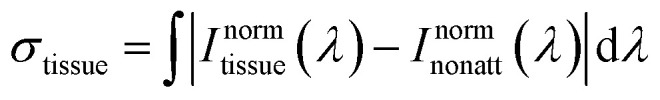
where *I*_tissue_ and *I*_nonatt_ denote respectively the spectra obtained with and without tissue, and the index norm indicates that the spectra are normalized to the area (*i.e.*, 
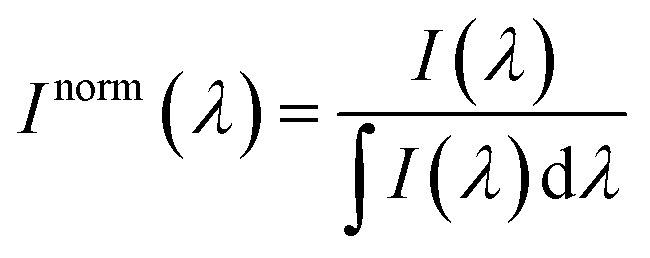
). The results included in Fig. 6(b), (d) and (f) reveal that the tissue-induced percentual change in the shape of the spectra in BW-I and BW-II are 20.5% and 21.6%, respectively (for a tissue thickness of 2 mm). On the other hand, the tissue-induced spectral distortion obtained in BW-III was significantly reduced down to 4%. When it comes to subcutaneous sensing based on ratiometric luminescence thermometry, the reduction of *σ* is of utmost importance, as it has been explained previously. Hence, we will hereafter focus on the emission of our CaF_2_:Yb^3+^/Er^3+^/Tm^3+^ NPs lying within BW-III for ratiometric thermal sensing.

**Fig. 7:**
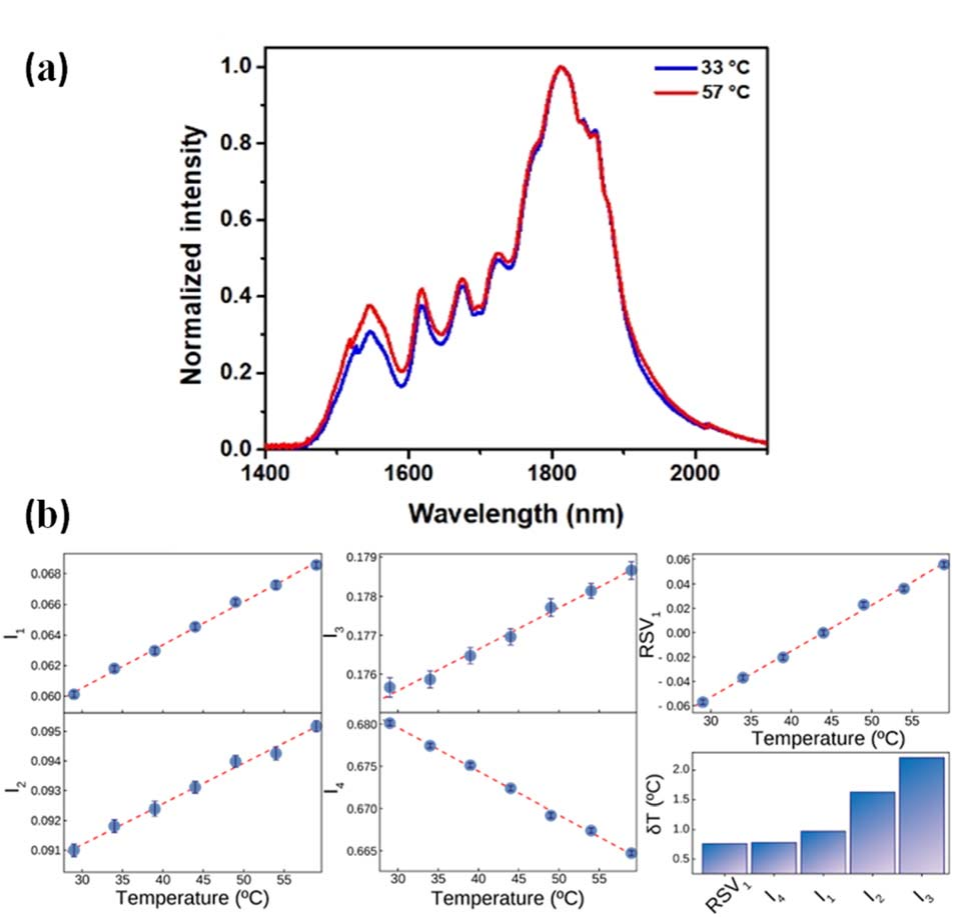
(a) Emission spectra of Yb^3+^/Er^3+^/Tm^3+^ CaF_2_ NPs at 33 °C (blue) and 57 °C (red) under 940 nm excitation in the III-BW. (b) Thermal dependence of the different normalized intensities (as defined in the main text) of RSV_1_ and the thermal uncertainties obtained with the different parameters.

Fig. 7(a) shows the normalized (to the intensity at 1812 nm) emission spectra of CaF_2_:Yb^3+^/Er^3+^/Tm^3+^ NPs as obtained at 33 and 57 °C in BW-III. A relative reduction in the emission of Tm^3+^ ions at around 1800 nm and an increment in the emission of Er^3+^ at around 1550 nm are observed. Due to the different thermal quenching of Er^3+^ and Tm^3+^ ions and to the population distribution between the Tm^3+^ levels, the shape of the emission spectra changes with temperature. This opens the possibility of using the BW-III band of CaF_2_:Yb^3+^/Er^3+^/Tm^3+^ NPs for thermal sensing. As it has been discussed previously, reliable ratiometric thermal sensing would be possible only by considering the emission lines that are within the spectral range in which the extinction coefficient is flat. Then, according to the data included in Fig. 2, reliable thermal sensing can be achieved by using the temperature dependence of the intensities of the emission lines lying within the 1590–1860 nm range. This means that for thermal sensing purposes we can use the temperature dependence of the emission lines at 1618, 1675, 1725 and 1812 nm normalized to integrated emitted intensity (*I*_1_, *I*_2_, *I*_3_ and *I*_4_, respectively). The temperature variations of these intensities (normalized to the integrated emission in the whole band) are shown in Fig. 7(b). The traditional approach for ratiometric thermal sensing consists of recording the temperature variation of these normalized intensities so that a proper analysis of emission shape can give the thermal readout. Alternatively, as recently demonstrated, multiple linear regression can also be applied to obtain a larger thermal sensitivity.^50^ Nevertheless, computing the thermal dependence of each of these parameters and building a regression model based on them can be a burdensome task. As a matter of fact, it can be a statistically complex process due to the possibility of the dependent variables being collinear. Thus, to avoid these issues and make the analysis simpler, we applied single value decomposition (SVD) to the dataset containing the temperature dependence of relative emitted intensities at these wavelengths. SVD is a computational method that falls into the category known as dimensionality reduction (DR), which, simply put,^51,52^ is a way of representing a dataset in a space of lower dimensionality. It is a matrix factorization technique widely used in scientific research and a powerful tool that can extract important information from large datasets, such as patterns and relationships between variables. SVD decomposes a matrix dataset into three other matrices: a left singular matrix, a diagonal matrix, and a right singular matrix. The diagonal matrix contains the singular values of the original matrix, which represent the relative importance of each column and row in the original matrix. These singular values can be used to reduce the dimensionality of the original matrix, allowing for easier computation and visualization of data. It has a wide range of applications in various fields, such as computer vision, natural language processing, and recommendation systems. It has several applications in spectroscopy, particularly in processing and analysing spectroscopic data. One of the main applications is noise reduction. Spectroscopic data often contain a significant amount of noise, which can affect the accuracy and reliability of the analysis. SVD can be used to remove noise from the data by identifying the principal components of the data and eliminating the components that are due to noise. This results in a cleaner and more accurate spectrum, which can be used for further analysis. For our purposes, SVD is a very handy technique since visualizing the simultaneous dependence of four different parameters is not so straightforward. Additionally, DR techniques have recently been shown to best summarize the calibration of a luminescent thermometer and to provide better precision in thermal readouts.^33^

When applying SVD to the dataset defined using all the relative intensities, more than 95% of the variance (computed from the relative contribution of the eigenvalues Fig. 7(b)) can be explained using a single vector (hereafter RSV_1_). Both the values of the level of variance explained and the coordinates of RSV_1_ are found through the factorization of the data matrix. The thermal dependence of RSV_1_ is included in Fig. 7(b) and represents a very linear trend. When estimating the thermal resolution achieved by the readouts given by the four relative intensities and the readout provided by RSV_1_, it has been shown that the uncertainty is minimized with the latter although the performance improvement with respect to that achieved by analysing only *I*_4_ is reduced (bar graph included in Fig. 7(b)).

## Conclusions

In conclusion, we investigated the potential of a luminescent nanothermometer based on Yb^3+^/Er^3+^/Tm^3+^ tri-doped calcium fluoride (CaF_2_) nanoparticles operating in three biological windows. It was shown that their emission in the third biological window was minimally affected by the presence of a biological tissue. Hence, it could be optimal for subcutaneous luminescence sensing. The thermal dependence of the luminescence of the nanoparticles was then studied and several thermometric parameters were identified. The application of singular value decomposition to the set of parameters provided a single parameter that could account for more than 95% of the variance in the calibration. Such a parameter, in turn, was shown to be linearly dependent on temperature and provided a minimum thermal uncertainty below 1 °C. In short, the use of BW-III is a great solution to the problem recently presented by the scientific community, recovering the reliability of luminescent thermometers for bio-applications and the tri-doped CaF_2_ matrix with Yb^3+^, Tm^3+^ and Er^3+^ is a luminescent nanothermometer with high relative thermal sensitivity.

## Author contributions

A. C. C. Soares and T. O. Sales contributed to conceptualization, data curation, formal analysis, investigation, visualization, and writing the original draft. E. C. Ximendes was responsible for data curation, formal analysis, methodology, validation, and writing – review & editing. Daniel Jaque contributed to funding acquisition, methodology and writing – review & editing. C. Jacinto was responsible for project administration, resources, supervision, funding acquisition and writing – review & editing.

## Conflicts of interest

There are no conflicts to declare.

## Supplementary Material
